# Comprehensive Physiotherapy Protocol in Post-operative Case of Trimalleolar Fracture: A Case Report

**DOI:** 10.7759/cureus.50705

**Published:** 2023-12-18

**Authors:** Ishika T Agrawal, Vaishnavi M Thakre, Maithili M Deshpande, Chinmay Bahirde

**Affiliations:** 1 Musculoskeletal Physiotherapy, Ravi Nair Physiotherapy College, Datta Meghe Institute of Higher Education and Research, Wardha, IND

**Keywords:** orif, exercise, rehabilitation, ankle fractures, physiotherapy, malleolus, trimalleolar fracture

## Abstract

A common ankle fracture that can have major consequences and expensive medical bills is the trimalleolar fracture. The trimalleolar fracture is the least frequent type of ankle fracture. The number of afflicted malleoli, the kind of fracture of the lateral and medial malleolus and the congruence of the ankle joint were all examined in detail for the trimalleolar ankle fracture. This type of fracture is brought on by high-energy trauma. In this case report, we describe a 56-year-old female patient who was involved in a road traffic accident. After being taken to the hospital for further examination, her ankle fracture was determined to be a trimalleolar one. She had an internal fixation with canulated cancellous screws and nails for open reduction. For such patients, we designed a physiotherapy course based on early rehabilitation and sensorimotor retraining to help with proprioception training, gait training and lower-limb muscular strength training. The lower limb's strength and range of motion were improved with the treatment. The outcomes used were the numerical pain rating scale (NPRS), functional independence measure (FIM), lower-extremity functional scale (LEFS), range of motion (ROM), gait parameters and manual muscle testing (MMT).

## Introduction

The most common kind of fracture in the lower extremities is an ankle fracture [1]. Such fractures are categorised as unimalleolar, bimalleolar or trimalleolar fractures according to the number of malleoli that are affected. A type of complicated ankle fracture known as a trimalleolar ankle fracture is composed of a bimalleolar fracture and a posterior malleolar fracture [2]. Henderson used the phrase 'trimalleolar fracture' for the first time [3]. The incidence rate is twice as common in females than in males [2]. Being the most severe type of ankle fracture, it invariably requires immediate surgical intervention for internal fixation and reduction. Women who are at primary risk of fall injuries are 75-84 years of age and have the highest incidence. Meanwhile, men in their 20s and 30s are susceptible to high-impact trauma [4].

Trimalleolar fractures are still highly challenging to reduce and treat. Patients typically experience a certain kind of sequelae after treatment, such as traumatic arthritis, joint deformity, gait instability and joint stiffness [5]. Rehabilitation programs are frequently offered to address these consequences. They can start soon after the fracture has been stabilised, at the time of the immobilization period or, more frequently, after the immobilisation period has ended, or when a bone union has occurred [6]. Early post-operative pain-dependent weight bearing and mobilisation improved range of motion, led to a quicker return to work and produced a better short-term Olerud-Molander Ankle Score (OMAS) [7].

Joint mobilisation is one of the physiotherapy methods for restoring range of motion. In Mulligan's idea of mobilisation with movement (MWM), a type of manual therapy in which the patient engages in an active movement while the therapist applies a gliding force. The objective of applied manual therapy is to rehabilitate the ankle joint as functionally as feasible, providing support to the lower limb to regain its original state [2]. The majority of studies assessed the functional state of patients following ankle fracture procedures using radiographic findings, physical examination and patient-reported outcome measures [8]. However, surgical intervention should be considered for patients who are older and have a significant medical history.

According to a 2001 prospective research, patients over 55 years of age who received open reduction and internal fixation had considerably better functional results and ankle range of motion than those who only had a closed reduction [4]. After surgery or after taking off of a plaster cast, rehabilitation starts. A specialised rehabilitation session that incorporates exercises for ankle mobility resisted exercise, weight-bearing and balancing is necessary due to the combination of pain, decreased muscle strength, altered proprioception and restricted active and passive range resulting from the fracture [9-11]. Restoring the lower limb's natural supporting function is an aim of applied manual therapy, with the objective being as the functioning of an ankle joint restoration as possible. Hence, we have established a physiotherapy protocol for patients who have undergone surgery to prevent post-operative complications due to immobility.

## Case presentation

Patient information

We present you a case of a 56-year-old female who met with a road traffic accident and was brought to an emergency causality, having a history of being hit by a vehicle from behind while standing on the road. She was brought to the hospital while unconscious, where her wounds were treated. X-ray and computed tomography (CT) were conducted as a part of examination. After two to three hours, she regained consciousness. The CT scan report was normal, while the X-ray revealed a trimalleolar fracture of the right ankle. Open reduction and internal fixation (ORIF) with a cannulated cancellous screw and rush nail were performed as a part of orthopaedic treatment. Moreover, for one and a half month, a plaster cast was applied from the mid-thigh to the midfoot. After 1.5 months, the cast was removed, and then the patient complained of pain, swelling and difficulty in performing day-to-day activities, including walking and climbing; thus, physiotherapy was started.

Clinical findings

The patient's informed consent was obtained verbally and in writing before the physical examination. The numerical pain rating scale (NPRS) indicated that the right ankle’s dull-aching pain was 4/10 during weight-bearing movement and 0/10 at rest. Grade 2 tenderness was present on both the malleolus, along with swelling on palpation. Girth measurement confirmed the muscle atrophy in the right calf muscle, showing a difference of 1.5 cm. A true leg length discrepancy of 1.5 cm has been observed in the affected extremity. The active and passive ranges of motion were restricted.

Radiological findings

**Figure 1:**
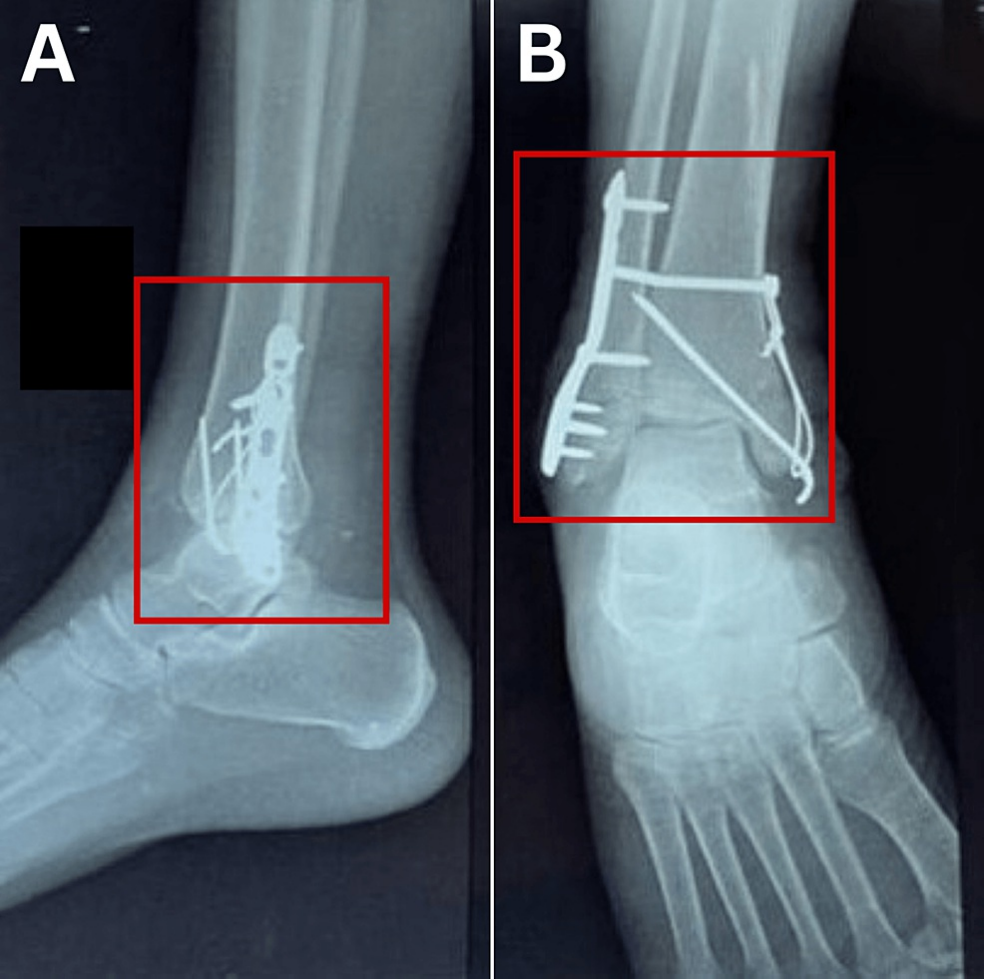
Lateral and anteroposterior (AP) views of the post-operative X-ray of the right ankle Figure 1A, 1B : Red-colored rectangle demonstrates the trimalleolar fracture, which was treated by open reduction and internal fixation (ORIF) along with a cannulated cancellous screw and rush nail in the lateral and anteroposterior views, respectively.

After surgery, radiographic examinations were carried out to observe how the implants were fixed, and it revealed a trimalleolar fracture, which was treated by ORIF along with a cannulated cancellous screw and rush nail (Figure 1A-1B).

Therapeutic intervention

**Figure 2:**
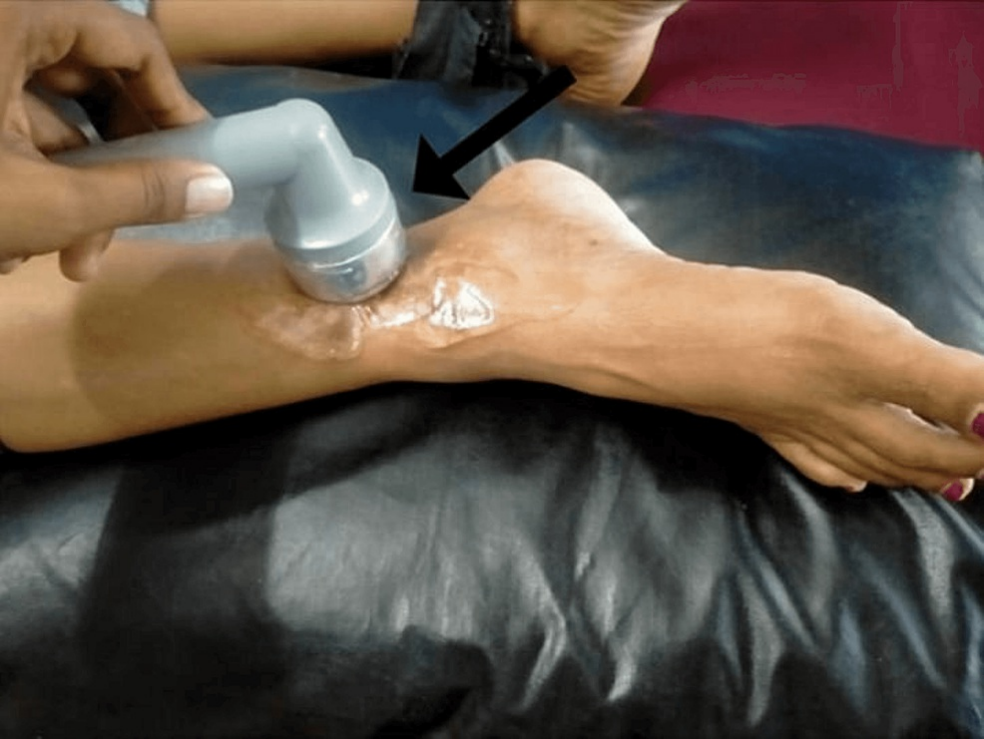
Patient undergoing ultrasound therapy for a scar mobilization

The objectives were pain reduction, increased lower limb strength, increased ankle range of motion, improved scar pliability, proprioception and improved gait training (Table 1 and Figure 2).

**Table 1:** Physiotherapy protocol NA: not applicable, ROM: range of motion, Reps: repetitions

Goal	Intervention	Dosage	Progression
Patient and family education	The patient and his family received a thorough explanation of his situation and the significance of physiotherapy intervention	NA	Home programme explained
To reduce swelling	Cryotherapy	7 minutes, twice daily	NA
To reduce pain (to increase blood flow and to eliminate metabolic waste)	Hot pack	15 minutes, twice daily	NA
To improve the pliability of scar tissue	Ultrasound	7 minutes at 1 watt/cm^2^ intensity, once daily; continuous mode	Reduce time and intensity
To release scar	Cross-friction massage	10 minutes, twice daily	NA
To avoid secondary consequences such as venous thrombosis	bilateral alternate dorsi flexion and plantar flexion of the ankle	20 reps × 1 set, every 4 hours	NA
To restore mobility of hip and ankle joints	Assisted ROM exercise of affected hip and ankle	10 reps × 1 set, twice daily	Active ROM exercise of affected hip and ankle
To improve flexibility	Stretching technique	10 reps with 10 seconds hold with band	Self-stretching with resistance band
To improve muscular strength of lower extremities	Isometric exercises of the quadriceps, iliopsoas, gluteus maximus, medius and minimus	10 reps × 1 set with 5 seconds hold	Dynamic resistance exercise
To improve proprioception training	Weight-bearing, weight shifts, stepping up-down, forward flexion, extension, abduction of affected leg	10 reps × 1 set	NA
To achieve equilibrium	Balance retraining in parallel bars	Twice daily	Crutches-based gait training leading to independent ambulation

Follow-up outcome measures

Following the 21 days of physical therapy, a follow-up was conducted by the same physiotherapist who treated the patient. Pre- and post-treatment values of various parameters are shown in Tables 2-3. The leg-length discrepancy was taken from the floor to heel in stance standing. In pre-rehabilitation, it was 4 cm, and after rehabilitation, it was 1.5 cm.

**Table 2:** Pre- and post-rehabilitation outcome measures cm: centimeter, Min: minutes

Ankle range of motion	Pre-rehabilitation	Post-rehabilitation
Dorsiflexion	0^0^-5^0^	0^0^-12^0^
Plantar-flexion	0^0^-20^0^	0^0^-25^0^
Manual muscle testing	Pre-gradings out of 5	Post-gradings out of 5
Hip flexors	3	4
Hip extensors	3	4
Plantarflexors	3	3
Dorsiflexors	2	3
Gait parameters	Pre-rehabilitation	Post-rehabilitation
Stride length	40 cm	50 cm
Step width	14 cm	17 cm
Step length	19 cm	24 cm
Cadence	76 steps/min	84 steps/min

**Table 3:** Pre- and post-intervention scales

Scales	Pre-rehabilitation	Post-rehabilitation
Numerical pain rating scale	4/10	2/10
Functional independence measure	25/126	110/126
Lower-exxtremity functional scale	20/80	65/85
Olerud-Molander Ankle Score	10/100	80/100

## Discussion

In this case report, we have discussed a case of a 56-year-old female suffering from a right ankle trimalleolar fracture managed by ORIF. Making the patient independent was the main objective of the physiotherapy management, and to that aim, a rehabilitation regimen was designed that comprised resisted exercise, increasing range of motion, proprioception training and improved gait training, also restoring joint mobility, regaining muscular strength, neuromuscular control and resuming pre-injury levels of activity [12]. More high-quality research needs to be done to assist patients suffering from ankle fractures. In this study, the effects of manual treatment were evaluated in patients who had ORIF, followed by extended immobilisation [13]. There have been findings that integrating balance, resistance exercise and proprioception training in rehabilitation sessions following an ankle injury might help with postural stability, coordination and proprioception [14].

Bhagwatkar et al. highlighted the effectiveness of rehabilitation for patellar fracture and medial malleolus fracture. The patient's rehabilitation was greatly aided by the early start of physiotherapy, which guaranteed the best possible functional recovery and reduced long-term difficulties. This instance highlights how crucial it is to use a multidisciplinary approach to treat patients with complex injuries. This approach should combine surgical skills with extensive physiotherapy therapies [15]. 

Analgesic effects and increased joint structure flexibility are produced by manual treatment techniques, such as joint mobilisation, which interact with the central nervous system, brain and body [16]. The study's conclusions, particularly with regard to discomfort and early movement, were consistent with those of earlier research [17]. Elderly people likely require different kinds of exercises or a longer training period in order to regain function. Certain factors that are known to affect functional recovery include motivation and self-efficacy [18]. Nilsson et al. used the OMAS to demonstrate that individuals over 40 were at risk for subjectively scoring poorly on function [19]. In the OMAS evaluation of subjective recovery, only minor disabilities have been reported in certain investigations [20].

## Conclusions

The 21-day physiotherapy protocol for a persistent post-operative triamellolar ankle fracture is described in this case report. The patient’s general functioning and ankle dorsiflexion strength improved under the care of physiotherapy, which comprised some mobilisation techniques, ultrasound therapy, resisted muscular, proprioceptive and gait training. The results of this case study highlight how crucial physical therapy exercises and interventions help patients perform activities of daily living.
